# Autoencoder based blind source separation for photoacoustic resolution enhancement

**DOI:** 10.1038/s41598-020-78310-5

**Published:** 2020-12-08

**Authors:** Matan Benyamin, Hadar Genish, Ran Califa, Lauren Wolbromsky, Michal Ganani, Zhen Wang, Shuyun Zhou, Zheng Xie, Zeev Zalevsky

**Affiliations:** 1 Faculty of Engineering and the Nanotechnology Center, Bar Ilan University, 5290002 Ramat Gan, Israel; 2 ContinUse Biometrics Ltd., Habarzel 32b Street, 6971048 Tel Aviv, Israel; 3 Key Laboratory of Photochemical Conversion and Optoelectronic Materials, Technical Institute of Physics and Chemistry, Chinese Academy of Sciences, Beijing, 100190 China; 4 University of Chinese Academy of Sciences, Beijing, 100049 China

**Keywords:** Imaging and sensing, Imaging techniques

## Abstract

Photoacoustics is a promising technique for in-depth imaging of biological tissues. However, the lateral resolution of photoacoustic imaging is limited by size of the optical excitation spot, and therefore by light diffraction and scattering. Several super-resolution approaches, among which methods based on localization of labels and particles, have been suggested, presenting promising but limited solutions. This work demonstrates a novel concept for extended-resolution imaging based on separation and localization of multiple sub-pixel absorbers, each characterized by a distinct acoustic response. Sparse autoencoder algorithm is used to blindly decompose the acoustic signal into its various sources and resolve sub-pixel features. This method can be used independently or as a combination with other super-resolution techniques to gain further resolution enhancement and may also be extended to other imaging schemes. In this paper, the general idea is presented in details and experimentally demonstrated.

## Introduction

Photoacoustic (PA) tomography is a multiscale imaging method, utilizing both minimal scattering of acoustic waves and excellent contrast of optical absorption. The lateral resolution in photoacoustics is determined by the specific configuration used; where acoustic resolution PA tomography (AR-PAT) is bounded by acoustic transducer’s bandwidth to few tens of microns^1^, optical resolution PA microscopy (OR-PAM) configurations reach better resolution, and are only limited by the size of the excitation spot. Deeper within the media, minimal spot size is increasing due to heavy scattering, and the resolution decreases accordingly. As a result, features smaller than the size of the excitation spot cannot be resolved.

Several methods for acquiring enhanced resolution PA images have been suggested in both acoustic and optical configurations. Label free approaches (by Yao^2^ and Danieli^3^), special known^4^ or random^5,6^ spatial excitation patterns, are common, however, most of them are somewhat limited to shallow depths within the tissue^7^. Particle localization is a wisely used solution for photoacoustic super-resolution, both AR-PAT^8–10^ and recently for OR-PAM^7^. However, localization requires some degree of spatial sparsity of the particles—a dense particle cluster eliminates the ability to determine the exact location of each particle within the illuminated area. In order to ease this requirement, multiple types of particles can be used, and a localization image can then be generated for each separately. This work presents a way to use multiple particle types for OR-PAM simultaneously and generalizes the use to as many particle types as possible (limited only by production considerations). In addition, in this work there is no need to rely on previously measured characteristics of the particles, unlike some suggested methods, instead, each particle should have a distinguishable signature. A localization process is applied to each particle type, and an enhanced resolution image is generated by superimposing all localized particle images. This concept can be used in combination with most of the existing label-based methods. Moreover, the technique may apply to both diffraction-limited excitation in the quasi-ballistic regime, but also to enhance resolution deeper inside the tissue in a quasi-diffusive regime, and thus extend the depth-of work of certain super-resolution techniques.

In some cases, such as photoacoustic tomography based on blood absorption, using contrast agents as nano-particles is unnecessary, However, since many OR-PAM applications, such us single cell microscopy^11^, Microvasculature imaging^9^, cancerous tumors imaging^12^ and many more, are already employing the injection of labels, adding more particle types and applying the suggested concept may be counted as a reasonable effort required to achieve resolution enhancement.

## Theory

The fundamental concept of the presented technology is the ability to unmix the responses of multiple particles excited at once. This signal can be unmixed in a few different ways. If there is a prior knowledge regarding the spectral response of each particle separately, the signal can be decomposed using this knowledge, this idea was previously purposed^13^. However, this work will focus on separating the particles’ responses without any prior knowledge, an approach commonly known as blind source separation. Blind source separation is a well-known problem in the field of statistical algorithms and machine learning^14,15^, vastly applied to acoustics^16^, neurophysiology^17^ and many other fields. The challenge faced by source separation is the decomposition of a given set of signals, mixed in an unknown manner, into their independent sources. Algorithms for separating sources are commonly relying on statistical independence of the sources (or sparsity in a different dimension), in order to calculate unmixing transformation matrix. The problem can be formalized as:

1 $$S = W_{s} X$$

where S is a matrix whose rows are signals sparse in a certain domain, $X$ is the observation signal matrix and $W_{s}$ is an unmixing matrix. We would like to find a $W_{s}$ such that:

2 $$W_{s} = \mathop{\arg \min}\nolimits_{{W_{s} }} \left| {X - W_{s}^{T} S\left| { = \arg \min_{{W_{s} }} } \right|X - W_{s}^{T} W_{s} X} \right|$$

That is, the error between the reconstruction, $W_{s}^{T} S$, and the source, $X$, is minimal. If we want the rows of $S$ to be independent, we may add:

3 $$W_{s} = \mathop{\arg \min}\nolimits_{{W_{s} }} \left| {X - W_{s}^{T} W_{s} X} \right| + \left\| {W_{s} } \right\|_{1}$$

This means that $W_{s}^{T} S$ and $X$ are roughly similar, so $W_{s}$ is decomposing $X$ into its independent sources. The minimization process is done using a gradient descent scheme. This solution to the unmixing problem, is commonly referred to as sparse autoencoder. In our case, in each excitation of a single pixel $\left( {r,c} \right),$ multiple particles respond together, creating a combined signal, $s_{p} \left( {r,c,t} \right):$

6 $$s_{p} \left( {r,c,t} \right) = a_{1} P_{1} \left( t \right) + a_{2} P_{2} \left( t \right) + \cdots + a_{N} P_{N} \left( t \right)$$

where $P_{n} \left( t \right)$ is the signal emitted from a single particle of type $n$ and $a_{n}$ are unknown weights related to the number of particles of type n within the excited area. If the source separation process is optimal, the rows of the sparse representation $S$, are the signals of each particle type separately. The mixing matrix, $W^{T}$ , reveals the relative weight of each particle within the signal of every pixel:

7 $$s_{p} \left( {r,c,t} \right) = w_{r,c,1}^{T} P_{1} \left( t \right) + w_{r,c,2}^{T} P_{2} \left( t \right) + \cdots + w_{r,c,N}^{T} P_{N} \left( t \right)$$

**Figure 1:**
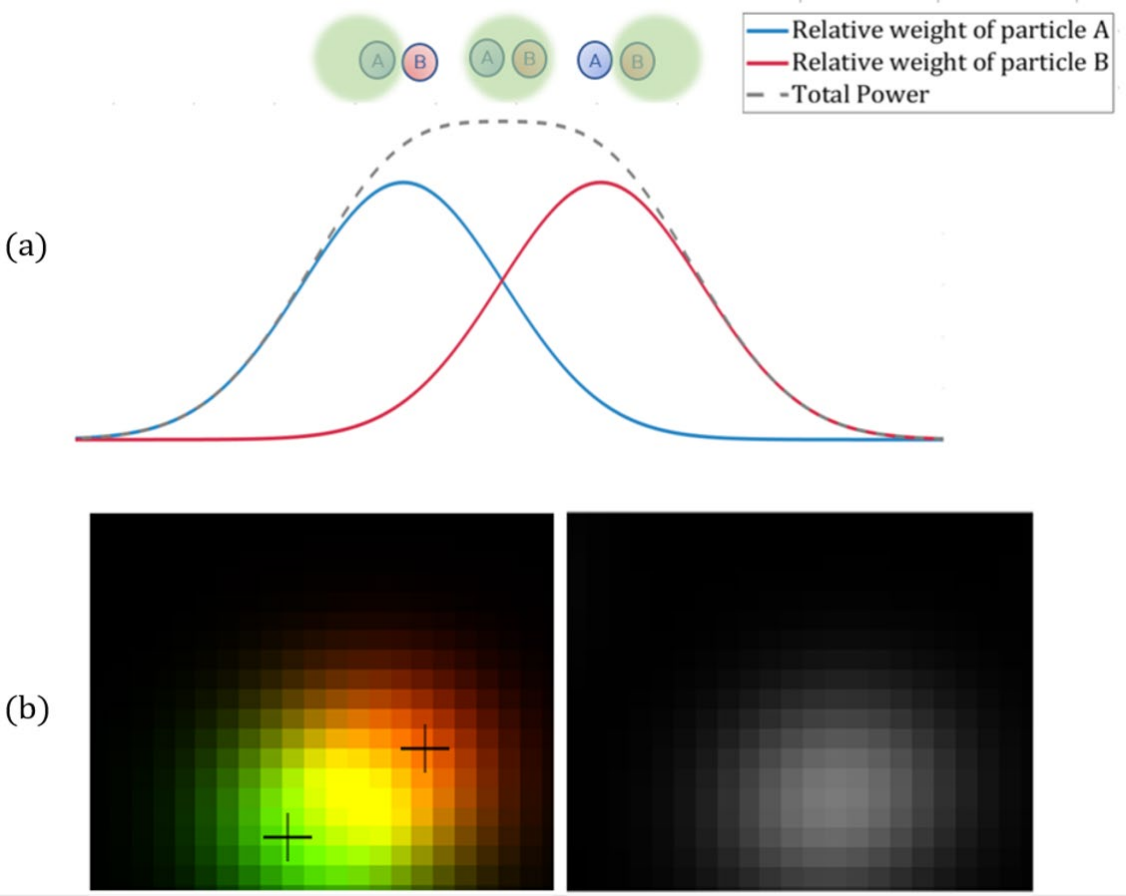
Multi-particle localization vs. standard localization. (**a**) The two particles A and B, excited by the green spot, cannot be resolved from the intensity profile (dashed black). However, the relative weights of A and B, derived from $W^{T}$, can be used to resolve the two particles, as they have maximas in different pixels. (**b**) A 2D demonstration of the idea. to the left, mixing matrix $W^{T}$ of two different particles, each presented in a different color (green, red). On the right, intensity image of the same two particles. Standard localization cannot distinguish between the particles.

While $W^{T} = \left( {w_{r,c,n}^{T} } \right)$ is the mixing matrix and its coefficients. Relative weight assists to resolve different types of particles even when they are closer to each other than the width of the point spread function (PSF), as further clarified in Fig. 1.

The full reconstruction procedure is as follows: (a) a signal is recorded for each excitation position, i.e., a pixel. The recorded signal at each pixel is a mixture of signals originating from many different particles present in the excited area. (b) the recorded signals are fed into a sparse autoencoder to generate a mixing matrix $W^{T}$. $W^{T}$ is a set of $N$ images ($N$ is the number of particle types), each denoted $W_{i}^{T}$. An image $W_{i}^{T}$ describes the relative donation of particle type $i$ to the signal of each pixel. (c) for each image $W_{i}^{T}$, the exact location of particle type $i$ is determined and localized in the peaks of $W_{i}^{T}$, and a new image is received, in which the particle is localized to only one pixel. (d) the $N$ localized images are summed and multiplied by the signal total intensity at each pixel.

## Methods

**Figure 2:**
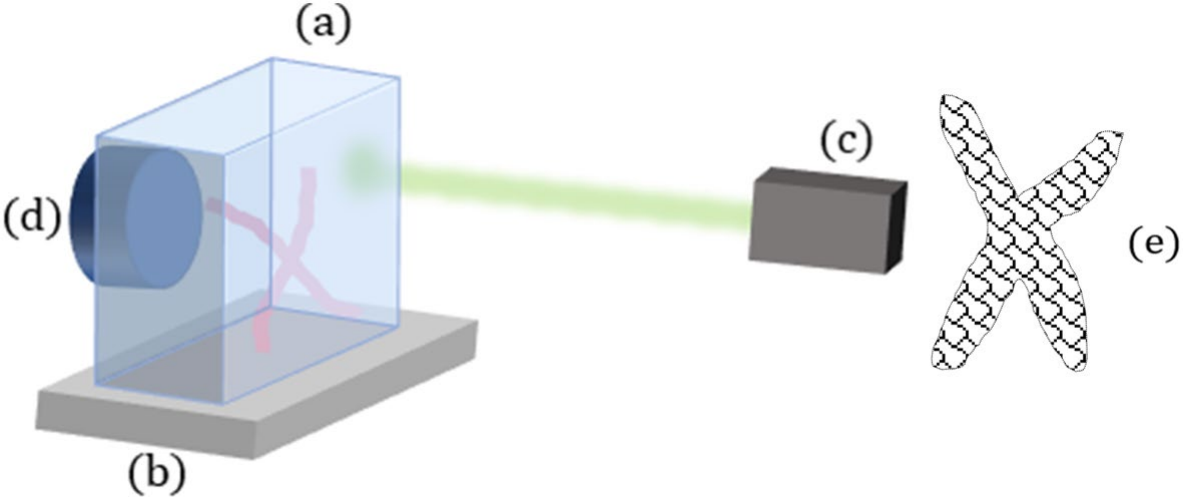
Experimental setup. A wax gel phantom (**a**) is put onto a moving stage (**b**). Photoacoustic excitation laser (**c**) excites the particles with a beam significantly larger than the width of each particle bubble. The signal is recorded using a piezo-electric transducer (**d**). A demonstration of phantom sectioning into bubble divisions (larger particles) is seen in (**e**).

A key factor in the purposed technique is that each particle type cannot appear more than once in the same excitation area. Otherwise, it will be impossible to resolve the two close particles (since they are of the same type) and the resolution will be reduced. In order to ensure that each particle type will mostly appear once in each excited area, it can be achieved in two ways—either use relatively large particle size compared to the size of the spot, or use a large number of types of small particles, so a large spot will statistically contain no more than one particle per type. In this paper due to reasons of time consumption and production ability, we chose to demonstrate the idea by using a model simulating large particles. Since the actual size of the particles is small (tens of nm), we simulated large particles by dividing the phantom into sections (bubbles) as demonstrated in Fig. 2, where each section represents a particle of this size. The sections were randomly filled with nano particles (the type of the particle was selected randomly). This way, the excitation spot mostly contains 1 or 2 bubbles of the same type.

It is important to emphasize that the most important requirement for this technique to work is the presence of one particle of the same type in each excitation spot, and this could be achieved, for a given excitation spot size, by either using many particle types or using less particle types but a model of large particles, as performed in this work. Both ways ensure to limit the number of particle types in each excitation spot, and therefore are equivalent, regarding the math describing the technique, as the fundamental issue is not the size of the particles or the total number of types, but rather the number of particle types in each excitation spot. A model simulating large particles (while actually using nanoparticles) could be simply achieved by creating divisions in the phantom, mimicking large particles. Since the exciton spot is larger than the size each ‘bubble’, all the particle in the bubble respond together, and therefore their effective size is the size of the bubble, not the particles inside it. While using multiple particle types is the applicative goal of this method, the technique itself could be demonstrated in a more simple and clear way by using a model of large particles. Nevertheless, further experiments of this concept will include demonstrating the same technique with small particles and more particle types, instead.

As a control experiment, all bubbles were filled with particle of only one type, demonstrating the current technology in PA and super-resolution photoacoustics.

**Figure 3:**
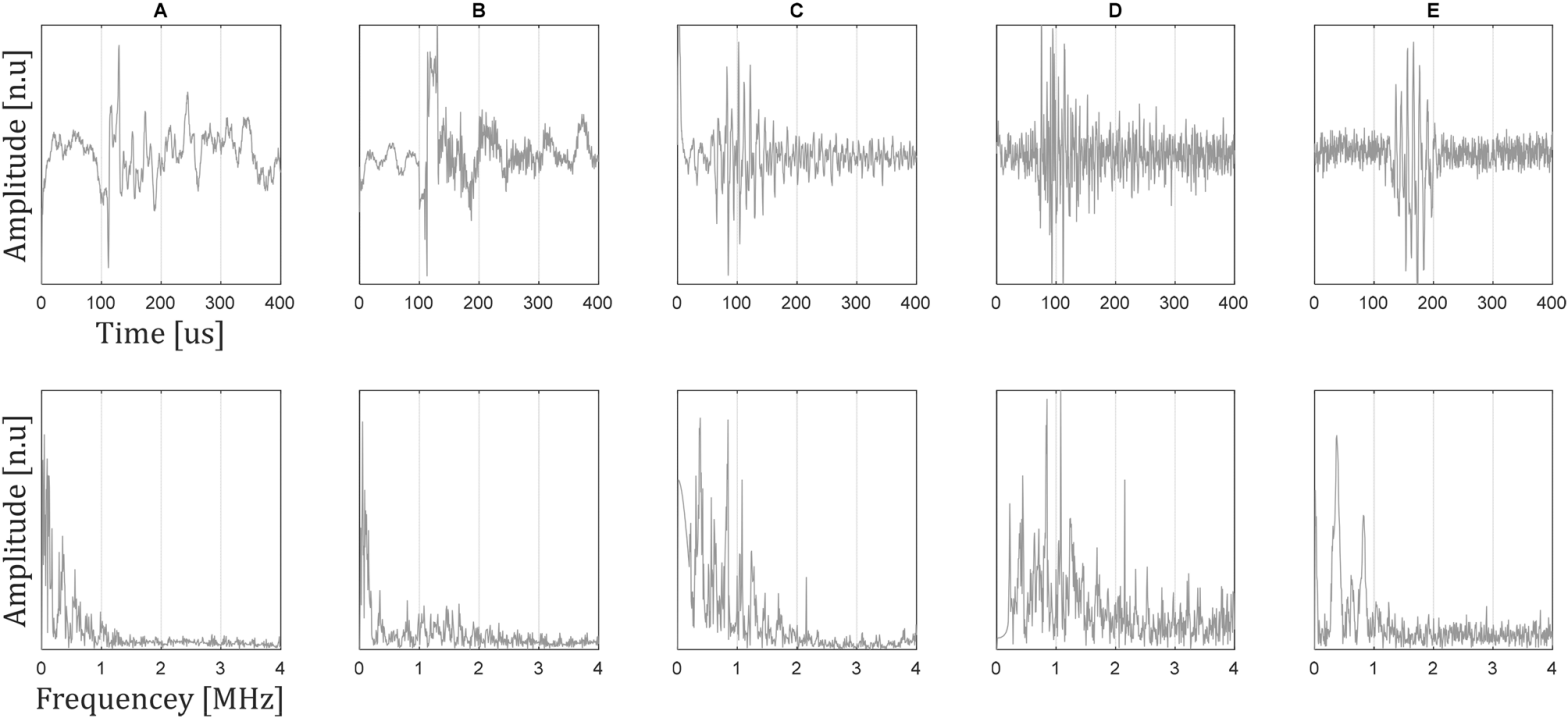
Signals recorded from each particle type separately. The signals were recorded using the piezo electric transducer, while in each of the recording a single type of particle was excited. The lower row is the spectrum of the signals, correspondingly.

This setup included 5 different types of particles; 2 types of graphene quantum dots with absorption peaks in 540 and 430 nm, denoted A and B (generation process described by Yuan et al^18^), respectively, C, D and E— 20 nm gold nano particles (nanoHybrids) conjugated with boronic acid and different concentrations of sialic acid (0, 5 and 15 mM respectively). Sialic acid is known to have a strong effect on the photoacoustic response of the particle^19^. For reasons of conciseness, the process of particle generation is not detailed here (since the use of specific particles is largely irrelevant—a combination of any particle type may be used for this method). However, it can be easily found in the references. The concentration of the particles was 1.5 $\frac{{{\text{mg}}}}{{{\text{mL}}}}$ for all particle types. The response of each particle type separately was recorded. In this demonstration of the method, the sparsity of the particles was expressed as difference in PA response bandwidth; however, as shown in Fig. 3, even though the spectra of the signals is different, there is no distinct well-defined way to differentiate between some of them (especially between particles B and C, or D and E) to the naked eye. The presented algorithm, on the other hand, has managed to differentiate between them, as will be shown. The model was a wax gel (Mindset) phantom, simulating a blood vessel bifurcation. The bubbles were created in a $5 \,\left[ {{\text{mm}}} \right]$ depth within the model, and the vessel had a diameter of $3 \,\left[ {{\text{mm}}} \right]$, and the average distance between the two vessels after the bifurcation is $5 \,\left[ {{\text{mm}}} \right]$. This vessel was divided into $400\, \left[ {\upmu {\text{m}}} \right]$ sized sections, each filled with a different particle type solution (randomly selected).

The excitation laser was a 532 nm 10 ns pulsed laser (Litron LPY 704G), with a beam diameter set to $1.5\, \left[ {{\text{mm}}} \right]$ using a $135\, \left[ {{\text{mm}}} \right]$ lens (Thorlabs). A scheme of the setup is demonstrated in Fig. 2. The large spot size, relative to the size of the imaged features makes conventional photoacoustic image incapable of differentiating between the vessels. The model was put onto a moving stage with step size $dx$ = 50 µm, and for each position of the stage, the photoacoustic signal was recorded using a 4 MHz central frequency piezoelectric transducer (Olympus CN4R-24). The signal from all excitation positions was decomposed using sparse autoencoder and a resolution enhanced signal was generated as described.

The autoencoder algorithm was a model of a learnt matrix, with dimension of $MXN$ where $M$ is number of pixels in the image (also the number of excitation positions) and $N$ is number of particles, in our case, 5. The elements of this learnt matrix were found via stochastic gradient descent algorithm, and the cost function used is described in Eq. (3).

In order to generally quantify the amount of sparsity required from the particles, a numerical simulation was performed. A synthetic image of capillary blood vessels was used as a phantom for the simulation. Into the capillary sample, 5 types of particles were randomly injected. The photoacoustic response of each of the particle types in the simulation stage, was set to be one of the real signals previously recorded in the experimental stage. as its photoacoustic response. However, since this stage explores sparsity, the bandwidth of each signal was tuned as desired, by linearly scaling the signal in the Fourier domain (to values described below). The temporal resolution of the simulation was 0.1 µs and the spatial was 0.1 $\frac{{\upmu {\text{m}}}}{{{\text{pxl}}}}$. The excitation beam was a gaussian beam with waist of 20 µm, and step size of 0.5 µm in each direction (x and y). Those signals, along with the excitation and detection profile were put into the k-Wave photoacoustic simulation environment^20^ in MATLAB. The output of the simulation was a set of signals, each originating from exciting a different pixel, followed by image generation using our purposed method. Multiple images were created, in each one a different sparsity between the particles was used, where sparsity in this case is the difference between the narrowest bandwidth of particle type 1 and the widest bandwidth of particle type 5. The sparsity ranges between 200 kHz and 1 MHz in 20 kHz steps. A comparison between the various images, resembling the performance of the algorithm dealing with different sparsities of the particles, was estimated by calculating the mean absolute error between the original capillary image and the constructed image (both images were normalized). It should be noted that the simulation stage demonstrates the sensitivity to particle sparsity, specifically for the simulated setup, which is rather different that the setup presented in the experimental stage and nay be affected differently from bandwidth. However, the general behavior of the reconstructed image in different sparsities can be understood from the simulation.

## Results

Figure 3 presents the responses of each of the particle types separately. A morphological difference between the responses is visible, and this difference eases the process of blindly separating them from a mixed signal. However, a meaningful similarity between some of the spectra is visible, making it difficult to distinguish between the particles without using a suitable algorithm, as previously explained.

**Figure 4:**
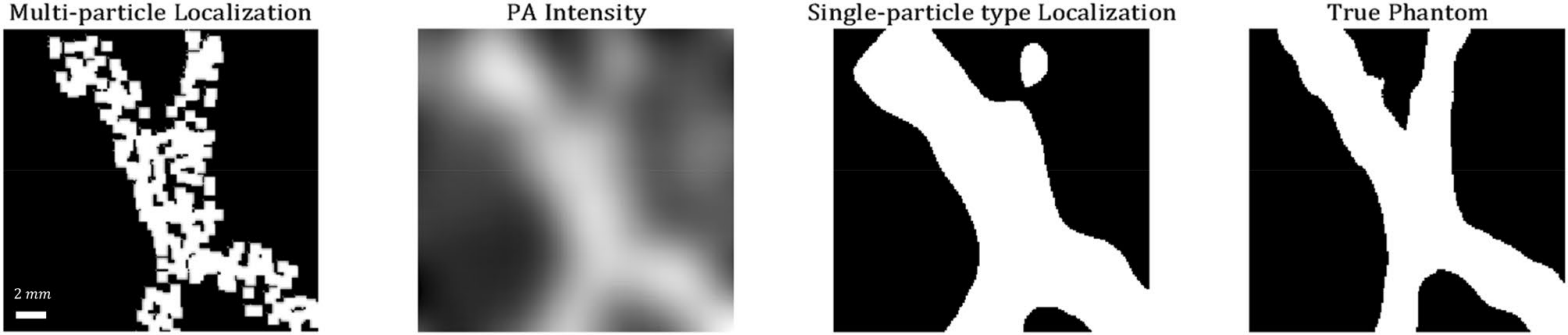
Imaging results. On the right, an image of the true 2 vessel phantom (the white area describes the area injected with particles). The second image from the right presents the localization-based image, not considering the various particle types. The second image from the left is the image reconstructed using photoacoustic intensity regardless of particle type (the traditional reconstruction scheme). The leftist image describes the multi-particle image reconstruction presented in this work.

In Fig. 4 the imaging results are presented. This figure describes a comparison between the multi-particle method suggested, and two conventional PA approaches—an image based on the PA intensity distribution (simply taking the intensity at every excitation pixel), and a localization scheme based on only one particle type (using a previously suggested 2-d gaussian fit localization scheme^7,8^) . Even though the 1-type image presents sharper edges than the intensity image, it is clearly much less detailed than the 5-particle image, especially around the bifurcation area, where the splitting of the two vessels is almost invisible in the 1-particle image. The multi-particle image has some black spots, indicating that the signal in this pixel did not correlate good enough to any of the particles (the relative weight of all particle types was equal).

**Figure 5:**
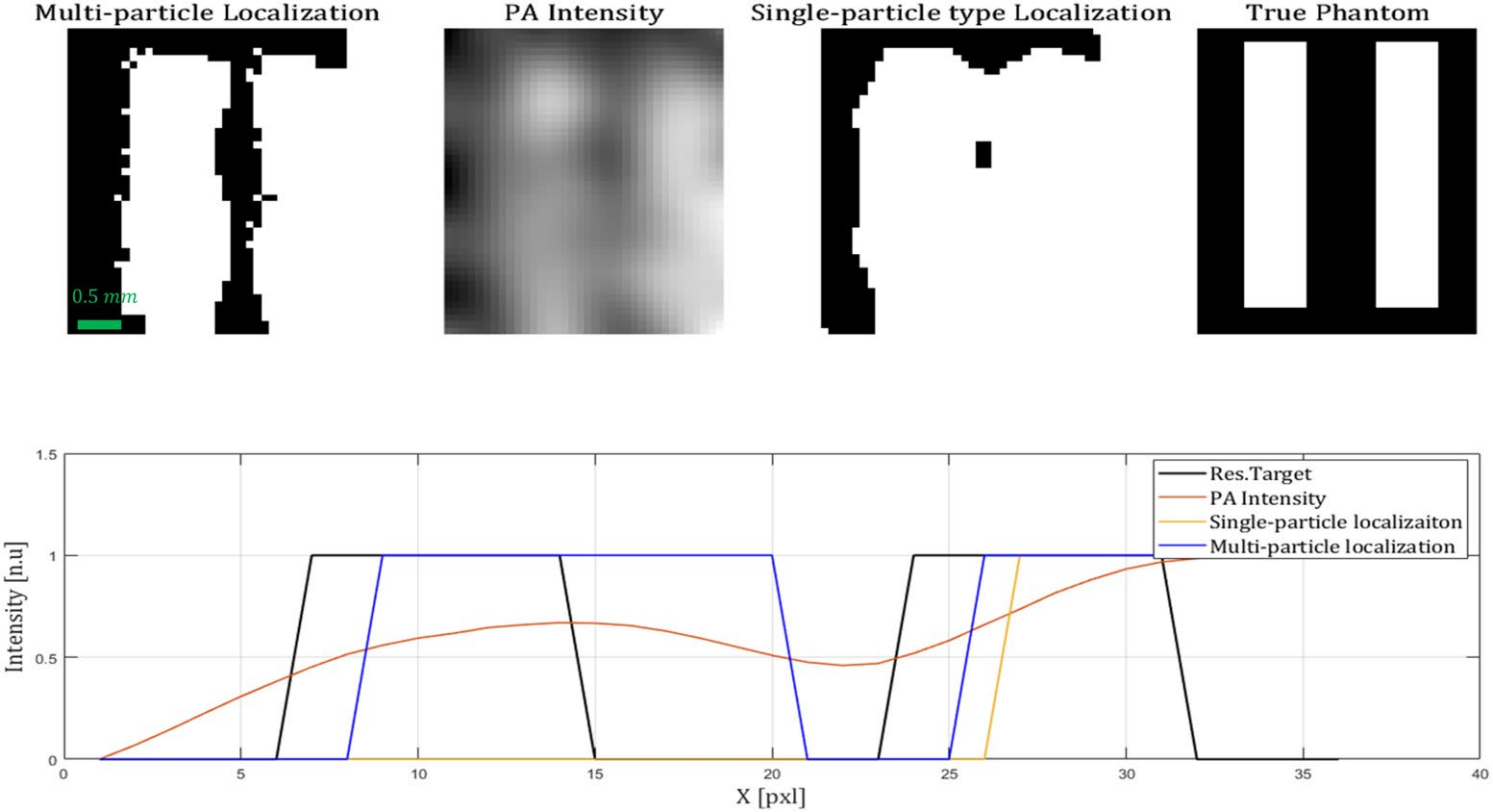
Resolution target. The real phantom, as well as the three reconstruction methods described above, are presented. The lower image describes a single line from the middle of each image, demonstrating the resolution ability.

In Fig. 5 the phantom used was a resolution target, to quantify the resolution enhancement. The width and distance between the bars were 0.7 [mm] (2–3 particle bubbles), where the size of the excitation spot and the step size were the same as the first experiment. It can be seen that the resolution enhancement factor in this experiment is only 2–3 (considering resolution is determined by the spot diameter). This can be a result of different particle distribution, since as mentioned the distribution of particle types is randomly selected. If two adjacent sections were to be filled with the same particle type, this would lower the resolution. However, even in this case the resolution was enhanced significantly.

**Figure 6:**
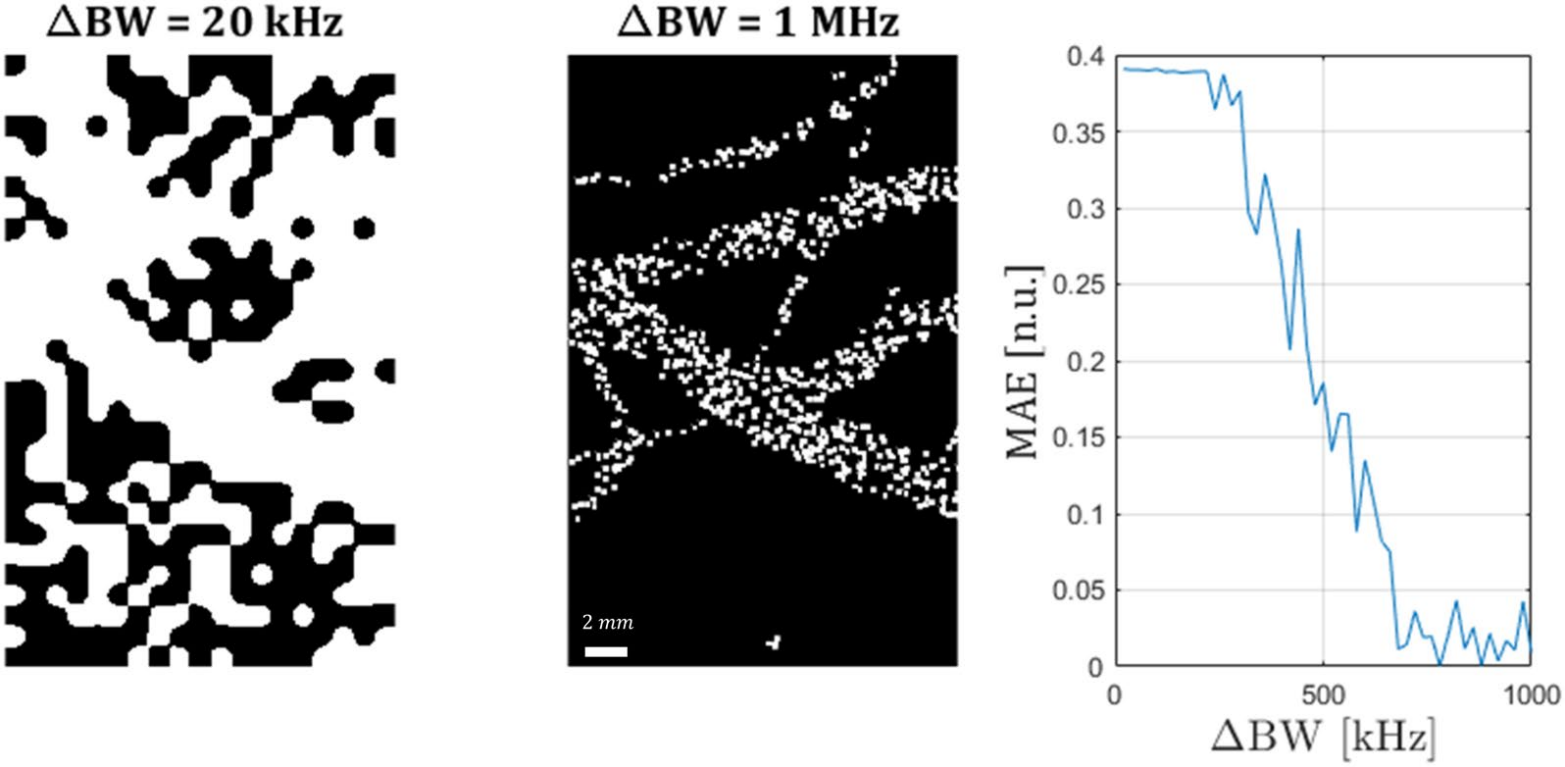
Bandwidth sparsity requirement*—*simulation results. In the left image, the worst sparsity (20 kHz difference of bandwidth) is presented. In the middle, the image with sparsity of 1 MHz is presented. The graph on the right demonstrates the mean average pixel error (MAE) as function of sparsity.

Figure 6 presents the results of the bandwidth sparsity requirement simulation. The left image is the image taken with the worst sparsity—20 kHz difference of bandwidth between the narrowest and the widest particle. In the middle, the image with sparsity of 1 MHz is presented. The graph on the right demonstrates the average pixel error as function of sparsity. It can be seen that in very low sparisties (< 200 kHz) the image is rather poor, and above ~ 600 kHz of bandwidth difference there is saturation in the quality of the image, as the signals are sufficiently separable. It should be noted that this simulation result only applies to the specific settings, number of pixels and particle sizes.

## Discussion

The feasibility of multi-particle localization concept has been successfully demonstrated. The experimental stage has demonstrated the theoretical resolution improvement factor of 5, by being able to discriminate two vessels with a distance five times smaller than diameter of the illumination (a situation which cannot have been achieved using either conventional OR-PAM nor localization based super resolution methods). In reality, the resolution improvement factor is affected by both the number of available particles, their sparsity, and the performance of the separation algorithm. The bandwidth of the particles in the experiment was between 200 and 800 kHz, a range of 600 kHz, which is more than sufficient according to the results of the simulation. A range of ${\Delta }BW = 200\,{\text{kHz}}$ for 5 particles means an average of 40 kHz per particle, so theoretically, using a modest 4 MHz transducer will allow 10 types of particles.

Future work will focus on in vivo investigation of the resolution improvement, as well as examining methods to reduce the sparsity level required from the various particles' responses. In addition, implementing the idea in AR-PAT configurations and non-scanning schemes may be also tested. The results demonstrate both the novelty and potential of the general idea, and the strength of using sparse autoencoder as source separator, in order to implement the idea as a standalone resolution enhancement tool, as well as an addition and enhancement for many other existing photoacoustic super-resolution methods.
